# Assessment of Acute Rejection by Global Longitudinal Strain and Cardiac Biomarkers in Heart-Transplanted Patients

**DOI:** 10.3389/fimmu.2022.841849

**Published:** 2022-03-25

**Authors:** Tor Skibsted Clemmensen, Nilufar Firooznia, Fariha Morsal Olawi, Brian Bridal Løgstrup, Steen Hvitfeldt Poulsen, Hans Eiskjær

**Affiliations:** ^1^ Department of Cardiology, Aarhus University Hospital, Aarhus, Denmark; ^2^ Institute of Clinical Medicine, Aarhus University Hospital, Aarhus, Denmark

**Keywords:** heart transplantation, acute rejection, echocardiography, speckle tracking imaging, cardiac biomarker, donor-specific antibodies

## Abstract

**Aims:**

The aim of this study was to evaluate left ventricular global longitudinal strain (LVGLS), N-terminal pro brain natriuretic peptide (Nt-ProBNP), and Troponin T as non-invasive markers for acute cellular rejection (ACR) diagnosis and severity assessment after heart transplantation (HTx).

**Methods:**

We retrospectively included all HTx patients transplanted from 2013 to 2019. At each visit, the patients were subjected to endomyocardial biopsy (EMB), measurement of Nt-ProBNP and Troponin T, and protocoled echocardiography with assessment of LVGLS. Sudden drop in graft function (SDGF) was defined as a drop in LVGLS ≥-2% in combination with either an increase in Troponin T ≥20% or Nt-ProBNP ≥30% compared with levels at the latest visit.

**Results:**

We included 1,436 EMBs from 83 HTx patients. The biopsies were grouped as 0R (n = 857), 1R (n = 538), and ≥2R (n = 41). LVGLS was lower and Troponin T and Nt-ProBNP higher in the 2R group than in the 0R and 1R groups (LVGLS: -12.9 ± 3.8% versus -16.9 ± 3.1% and -16.1 ± 3.3%; Troponin T: 79 [33;230] ng/l versus 27 [13;77] ng/l and 27 [14;68] ng/l; Nt-ProBNP: 4,174 [1,095;9,510] ng/l versus 734 [309;2,210] ng/l and 725 [305;2,082], all p < 0.01). A SDGF was seen at 45 visits of which 19 had ≥2R ACR. EMBs showed ACR in 20 cases without SDGF. Finally, neither was SDGF seen nor did the EMB show rejection in 1,136 cases. Thus, the sensitivity of SDGF for ≥2R ACR detection was 49% (32–65) and specificity 98% (97–99). The positive predictive value (PPV) was 42% (31–55) and the negative predictive value (NPV) 98% (98–99). The diagnostic value improved in a sub-analysis excluding EMBs within 3 months after HTx, clinically interpreted false positive ≥2R ACR cases, and cases with ≥2R ACR who recently (<2 weeks) were treated with intravenous methylprednisolone due to ≥2R ACR (sensitivity 75% (48–93), specificity 97% (96–98), NPV 99% (99–100), and PPV 39% (27–52).

**Conclusions:**

Patients with ≥2R ACR have lower LVGLS and higher Troponin T and Nt-ProBNP than patients without 2R rejection. A non-invasive model combining changes in LVGLS and Troponin T or Nt-ProBNP showed excellent negative predictive value and moderate sensitivity and may be used as a gatekeeper to invasive biopsies after HTx.

## Introduction

Acute rejection remains a significant complication after heart transplantation (HTx) (1). Rejections occur predominantly during the first 6 months but can be seen at any time after HTx (2–4). Symptoms are uncommon in the early course, but eventually symptoms of heart failure or sudden cardiac death may occur. Endomyocardial biopsies are still the golden standard for detection of acute rejection (5, 6). However, the endomyocardial biopsy procedure is invasive and carries a well-known risk of complications such as pneumothorax, cardiac tamponade, lung embolism, and lesions on the tricuspid valve. Furthermore, rejections are often patchy and biopsies may not detect or underestimate the rejection severity. Moreover, the histological interpretation is difficult with significant interobserver variation. Therefore, non-invasive methods for the evaluation of acute rejection are highly desirable to improve the diagnostic setup and the severity assessment. Echocardiographic assessment of left ventricular global longitudinal strain (LVGLS) has been demonstrated to be reduced during rejection episodes and to improve during resolving of the rejection (7). However, the overall reported sensitivity (56%–87%) and specificity (75%–81%) for rejection detection by LVGLS are at most moderate (7–10). Elevated N-terminal pro brain natriuretic peptide (Nt-ProBNP), a non-invasive plasma marker of elevated filling pressure, has previously been associated with acute rejection (11, 12). Furthermore, the degree of rejection-induced myocyte necrosis may be reflected in plasma levels of cardiac Troponin T (13). However, the roles of combined assessment of LVGLS and cardiac biomarkers are unknown.

Thus, the present study aimed at evaluating LVGLS, Nt-ProBNP, and high-sensitivity Troponin T as non-invasive markers for diagnosis and severity assessment of acute rejection.

## Methods

### Patient Population

In this retrospective study, we included all patients receiving heart transplantation at Aarhus University Hospital, Denmark, from 2013 to 2019. A total of 86 patients were transplanted during this period. However, three patients died prior to first visit with LVGLS assessment and were not included. Thus, we included 83 patients subjected to a total of 1,476 endomyocardial biopsies during a follow-up of 24 months [15;25]. A total of 40 biopsies were not included in the analysis as these were taken without assessment of LVGLS or cardiac serological markers. None of these biopsies showed treatment demanding rejection.

All patients received induction therapy with anti-thymocyte globulin for the first 3 postoperative days. The standard immunosuppressive regime comprised a calcineurin inhibitor, tacrolimus, in combination with mycophenolate mofetil. All patients received high-dose methylprednisolone for the first 2 postoperative days followed by prednisolone (0.3 mgkg/day), which was gradually reduced during the first postoperative year. Steroid treatment was stopped in most patients during the second year after HTx.

The post-transplant routine rejection surveillance included endomyocardial biopsies and comprehensive echocardiography and blood samples including the cardiac biomarkers high-sensitivity Troponin T and Nt-ProBNP.

Demographic and clinical data were collected from electronic patient files. Biopsy results were collected from the histological reports.

### Endomyocardial Biopsy

Biopsies were taken by standard local hospital protocol using the right internal jugular vein or a femoral vein. Patients underwent routine biopsies at the first 2 postoperative years. Biopsies were scheduled weekly during the first 6 weeks, every 2 weeks until 3 months, every month until 6 months, and every 2 months for the rest of the first postoperative year. Biopsies were taken every 3 months beyond the first year until year 2. Afterward, biopsies were only taken, when rejection was clinically suspected. Biopsies were routinely examined for histological signs of antibody-mediated rejection (AMR) with examination for C4D and CD68 deposits in case of histological signs of AMR, clinical signs or symptoms, or *de-novo* donor-specific antibodies.

Experienced cardiac pathologists analyzed all biopsies. Acute cellular rejection (ACR) was histopathologically graded as no (0R), mild (1R), moderate (2R), and severe rejection (3R), according to guidelines of ISHLT (14). Acute cellular rejections ≥ 2R were treated with intravenous methylprednisolone 1 g daily for 3 days. In contrast, we did not routinely treat 1R rejection episodes with changes in immunosuppression.

### Donor-Specific Antibodies

Patients were routinely screened for donor-specific antibodies (DSA) between the first and second years after HTx using LABScreen^®^ Mixed and LABScreen^®^ Single Antigen (One Lambda Inc., CA, USA). HLA antibody specificities above 1,000 MFI were considered positive.

### Echocardiography

We used a commercially available ultrasound system (Vivid E9 or E95, GE Healthcare Horten, Norway) with a 3.5-MHz phased array transducer (M5S). Echocardiography was performed by trained sonographers within 24 h of the biopsy and always before medical treatment of rejection. The sonographers were instructed to optimize image quality for assessment of myocardial deformation by LVGLS. LVGLS was obtained by frame-by-frame tracking of speckle patterns throughout the left-sided myocardium in standard 2D cine loops. The region of interest was manually adjusted for optimal tracking results. We excluded segments with unacceptably low tracking quality due to poor image acquisition or artifacts. LVGLS was calculated as the average of peak longitudinal systolic strain in a 17-segment myocardial model (15). LVGLS was assessed when the tracking quality was adequate in at least 5 of 6 segments in each view. The higher negative value of strain equals a higher magnitude of strain.

### Statistics

Normally distributed data are presented as mean ± standard deviation (SD); non-normally distributed data are presented as median and interquartile range [IQR]. Categorical data are presented as absolute values or percentages. Histograms and Q–Q plots were used to check continuous values for normality of the data distribution.

Our analysis plan involved the following:

- Comparison of myocardial function and cardiac markers between three different biopsy score groups; 0R, 1R, and ≥2R. Between-group differences were assessed using a mixed model for continuous variables due to the unequal number of observations between patients. We used receiver operating characteristic curves to calculate the diagnostic value of continuing variables for acute rejection prediction. The optimal cutoff points were defined as the intersection points of sensitivity and specificity in the receiver operating characteristic curves.
- Serial characterization of LVGLS and cardiac biomarkers before, during, and after ≥2R rejection. We used repeated ANOVA analysis to compare between visit changes.
- Comparison of differences in myocardial function and cardiac biomarkers between patients with sudden drop in graft function with and without biopsy-proven rejection ≥2R and patients without sudden drop in graft function but biopsy-proven rejection ≥2R.

Sudden drop in graft function was predefined as a drop in LVGLS magnitude ≥-2% in combination with either an increase in Troponin T ≥20% or Nt-ProBNP ≥30% compared with levels at the patient’s last visit. Changes within the normal range (LVGLS ≥-18%, Troponin T <14 ng/l or Nt-ProBNP < 300 ng/l) were not considered as sudden drop in graft function.

p values < 0.05 were considered statistically significant. Analyses were performed using STATA (StataCorp LP, College Station, TX, USA).

## Results

### Demographics

Baseline characteristics are shown in **Table 1**. During follow-up, 28 patients (34%) had at least one ≥2R rejection episode. A total of 1,436 biopsies were included. The biopsies were grouped as 0R (n = 856), 1R (n = 537), and ≥2R (n = 43). In the 2R group, 41 patients suffered acute cellular rejection and two patients suffered antibody-mediated rejection.

**Table 1:** Baseline characteristics according to rejection groups.

	Baseline (n = 83)	12-month follow-up (n = 67)	p-value
Male sex, n (%)	64 (77)		
Age at HTx, years	54 [40–63]		
Reason for HTx			
* Cardiomyopathy, n (%)*	48 (58)		
* IHD, n (%)*	20 (24)		
* Congenital heart disease, n (%)*	9 (11)		
* Other, n (%)*	6 (7)		
Weight, kg	81 ± 17	84 ± 19	<0.01
Body mass index, kg/m^2^	25.7 ± 0.5	26.7 ± 0.6	<0.01
Hypertension, n (%)	33 (47)	53 (75)	<0.01
Sensitized prior HTx, n (%)	25 (30)		
*Medication*			
Prednisolone, n (%)	83 (100)	66 (99)	0.28
Ciclosporine, n (%)	0 (0)	4 (6)	<0.05
Tacrolimus, n (%)	82 (99)	64 (96)	0.24
Mycophenolate, n (%)	83 (100)	65 (97)	0.12
mTOR inhibitor, n (%)	5 (6)	17 (25)	<0.01

Data are presented as mean ± SD, percentages, or median and [IQR].

HTx, heart transplantation; IHD, ischemic heart disease.

### Rejection Groups and Prediction of Acute Rejection

**Figure 1:**
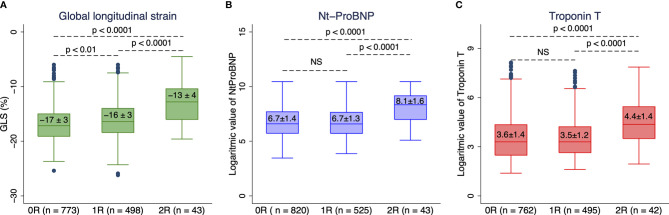
Boxplots comparing **(A)** echocardiographic global longitudinal strain magnitude (GLS), **(B)** plasma NT-ProBNP level, and **(C)** plasma high-sensitivity troponin T level between three biopsy groups (0R, no rejection; 1R, mild rejection; and ≥2R, severe treatment demanding rejection). NS, not significant.

**Table 2** shows hemodynamics, myocardial function, and serological markers in the three rejection groups. As depicted, systolic and diastolic blood pressure was slightly lower in the ≥2R rejection group than in the 0R and 1R groups and the trans-mitral Doppler flows revealed a more restrictive LV-filling pattern in the ≥2R group than in the 0R and 1R groups. LVGLS magnitude was significantly lower and Troponin T and Nt-ProBNP significantly higher in the 2R group than in the 0R and 1R groups. Notably, LVGLS magnitude was significantly lower in the 1R than 0R rejection groups (**Figure 1**).

**Table 2:** Hemodynamics, myocardial function, and serological markers according to rejection group.

	0R (n = 856)	1R (n = 537)	2R (n = 43)	Mixed-model p-value
***Hemodynamics***				
Heart rate, bpm	86 ± 12	87 ± 12	89 ± 17	0.26
Systolic blood pressure, mmHg	138 ± 15	138 ± 16	131 ± 18	0.01
Diastolic blood pressure, mmHg	85 ± 11	86 ± 11	81 ± 14	0.01
***Systolic function***				
LV-EF, %	60 ± 5	60 ± 5	56 ± 11	<0.0001
LV-GLS, %	-16.9 ± 3.1	-16.1 ± 3.3	-12.7 ± 3.7	<0.0001*
TAPSE, mm	14.6 ± 3.5	14.3 ± 3.4	12.9 ± 4.0	0.03
***Diastolic function***				
E/A ratio	2.0 ± 0.9	1.9 ± 0.6	2.3 ± 0.9	0.02
Deceleration time, ms	162 ± 32	163 ± 33	145 ± 37	0.02
IVRT, ms	80 ± 17	78 ± 16	81 ± 23	0.49
***Biomarkers***				
Creatinine, mmol/L	102 [81;130]	94 [77;117]	93 [72;122]	0.002*
Hemoglobin, mmol/L	7.3 [6.5;8.2]	7.5 [6.9;8.3]	6.6 [5.8;8.4]	0.09
Troponin T, ng/L	27 [12;76]	27 [14;68]	79 [32;232]	0.001
NT-ProBNP, ng/L	734 [307;2190]	713 [305;2082]	4174 [910;14448]	<0.0001

Mixed-model p-values using the mixed model to adjust for unequal number of observations per individual. Data are presented as mean ± SD, percentages, or median and [IQR].

*p < 0.05 comparing 0R versus 1R.

BPM, beats per minute; LV, left ventricular; EF, ejection fraction; GLS, global longitudinal strain; TAPSE, tricuspid annular plane systolic excursion; IVRT, isovolumetric relaxation time.

**Figure 2:**
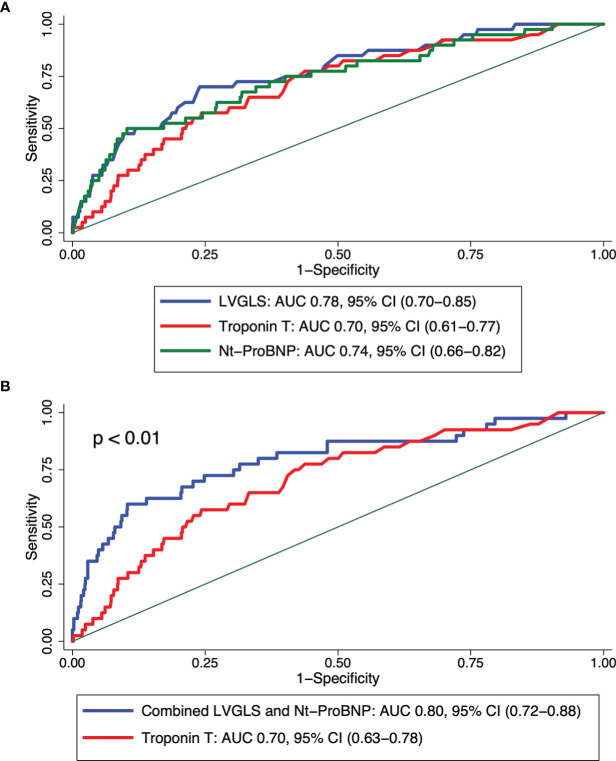
Receiver operating characteristic (ROC) curves demonstrating **(A)** the ability to predict severe acute rejection (≥2R) by echocardiographic left ventricular global longitudinal strain (LVGLS) magnitude, plasma NT-ProBNP level, and plasma high-sensitivity troponin T level and **(B)** the ability to predict severe acute rejection (≥2R) by combined echocardiographic left ventricular global longitudinal strain (LVGLS) magnitude and plasma NT-ProBNP level versus plasma high-sensitivity Troponin T level to predict ≥2R rejection.

The abilities of LVGLS, Troponin T, and Nt-ProBNP to predict ≥2R acute rejection are depicted in **Figure 2**. The normal range cutoff points for rejection prediction by LVGLS (<-18%), Troponin T (>14 ng/l), and Nt-ProBNP (>300 ng/l) provided high sensitivity (88.4%, 92.3%, 95.4%, respectively) but low specificity (35.3%, 26.3%, 24.2%, respectively). The optimal cutoff points for rejection prediction were LVGLS: -14.9% (sensitivity 72% and specificity 73%), Troponin T: 43 ng/l (sensitivity and specificity both 64%), and Nt-ProBNP 1,553 ng/l (sensitivity and specificity both 67%). By combining LVGLS, Troponin T, and Nt-ProBNP in a logistic model, the diagnostic accuracy increased providing AUC of 0.80 (0.72–0.88).

In a logistic model, LVGLS [odds ratio (OR) 1.4 (1.2–1.5)], the logistic value of Troponin T [OR 1.5 (1.2–1.8)], and the logistic value of Nt-ProBNP [OR 1.9 (1.6–2.4)] all predicted ≥2R acute rejection (all p < 0.0001). However, in a multivariate model, only LVGLS [OR 1.3 (1.2–1.4)] and the logistic value of Nt-ProBNP [OR 1.9 (1.3–2.7)] were independently associated with ≥2R acute rejection.

### Serial Changes in LVGLS and Cardiac Markers During and After Acute Rejection

**Figure 3:**
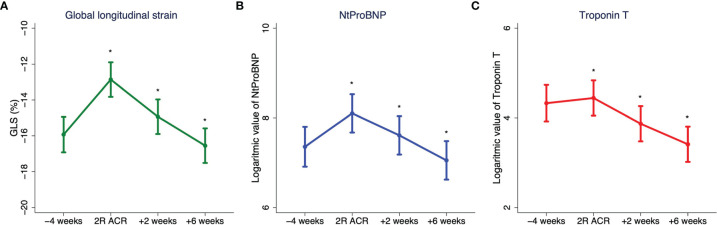
Margin plots with 95% confidence interval demonstrating changes in **(A)** echocardiographic left ventricular global longitudinal strain (GLS) magnitude, plasma NT-ProBNP level, and **(B)** plasma high-sensitivity Troponin T level **(C)** before, during, and after treatment demanding rejection (≥2R). “*significant difference (p<0.05) compared with previous visit.

**Figure 3** shows serial changes in LVGLS and the logarithmic value of Troponin T and Nt-ProBNP from the last biopsy before ≥2R rejection, the time of rejection, and the first two biopsies in the resolving phase afterward. As depicted, LVGLS magnitude significantly decreased and Troponin T and Nt-ProBNP significantly increased.

The median time from HTx to the patients last biopsy was 24 months [15;25]. At this time point, patients who had suffered at least one ≥2R rejection episode had a lower magnitude of LVGLS (-16.9% [-13.1;-18.8] versus -18.5 [-16.1;-19.6], p = 0.03) and higher Troponin T (19.0 ng/l [7.0;35.5] versus 12.0 ng/l [7;18], p = 0.02) and Nt-ProBNP (398 ng/l [226;1,278] versus 250 ng/l [111;583], p = 0.02) than patients who remained free from treatment demanding rejection.

DSA was detected in 12 patients between the first and second years post HTx of which 10 patients had HLA class II DSA and 2 patients had HLA class I DSA. The median MFI in patients with DSA was 1,800 [1,450;2,400]. Only 5 patients (18%) with acute rejection developed DSA whereas 23 patients (82%) did not develop DSA. Furthermore, we observed no difference in LVGLS (p = 0.90), Troponin T (p = 0.62), or Nt-ProBNP (p = 0.15) between patients with and without DSA development.

### Sudden Drop in Graft Function With and Without Acute Rejection

Based on predefined changes in LVGLS, Troponin T, and Nt-ProBNP, the biopsy visits were grouped as the following:

Group A) Sudden drop in graft function and biopsy-detected acute rejection (≥2R), n = 21

Group B) Sudden drop in graft function but no biopsy-detected acute rejection (≥2R), n = 24

Group C) No sudden drop in graft function but biopsy-detected acute rejection (≥2R), n = 20

Group D) No sudden drop in graft function and no biopsy-detected acute rejection (≥2R), N = 1,136

A sudden drop in graft function for detection of acute rejection (≥2R) showed a sensitivity of 51% (35–67), a specificity of 98% (97–99), a positive predictive value of 47% (35–59), and a negative predictive value of 98% (98–99). The overall accuracy of the model was 96% (95–97). The diagnostic performance increased in a subanalysis excluding biopsies taken within the first 3 months after HTx (number: group A = 14; group B = 1,714; group C = 8; group D = 671). Thus, we found sensitivity of 64% (41–83) and specificity 98% (96–99). The positive predictive value was 45% (32–59) and the negative predictive value 99% (98–99). Overall accuracy was 96% (95%–98%).

The clinical characteristics of group B are presented in **Supplementary Table 1**. As demonstrated, the vast majority of cases with a sudden drop of graft function without biopsy-proven rejection either were clinically interpreted as acute cellular or humoral rejection, had significant cardiac allograft vasculopathy, had decline in renal function, had acute arrhythmia, or were later diagnosed with acute rejection or suffered cardiac death during follow-up.

The clinical characteristics of group C are presented in **Supplementary Table 2**. Only 5/20 cases in group C had reduced (LVGLS <-18%). The biopsy result was clinically interpreted as false positive in three cases in which the patients did not receive intravenous methyl prednisolone. Notably, time since transplantation was significantly lower in group C (2.0 months [0.9;5.1]) than group A or B (4.9 months [1.3;23.3] and 6.6 months [2.3;18.6], respectively) (p < 0.0001).

In order to evaluate the changes in LVGLS, Troponin T, and Nt-ProBNP, we calculated the product of the absolute change and the percent increase or decrease. In a logistic model, the product of LVGLS change [odds ratio (OR) 2.4 (1.6–3.4)], Troponin T change [OR 3.5 (1.6–7.7)], and Nt-ProBNP change [OR 1.1 (1.1–1.2)] all predicted ≥2R acute rejection (all p < 0.001). However, in a multivariate model, only Nt-ProBNP change [OR 1.1 (1.1–1.2)] was independently associated with ≥2R acute rejection.

## Discussion

In this comprehensive study evaluating the relation between acute rejection, myocardial deformation, and cardiac biomarkers, several important findings were revealed: 1) acute rejections (≥2R) were rare and only observed in only 3% of biopsies; 2) patients with severe treatment demanding rejection (≥2R) have reduced LVGLS magnitude and increased Troponin T and Nt-ProBNP levels; 3) in the resolving phase after acute rejection, LVGLS improves and Troponin T and Nt-ProBNP decrease but remain elevated as compared to patients without acute rejection; 4) absolute isolated values of LVGLS, Troponin T, and Nt-ProBNP provide moderate sensitivity and specificity for acute rejection detection; and 5) changes in LVGLS and Troponin T and Nt-ProBNP provide excellent specificity and negative predictive value but at most moderate sensitivity for rejection detection.

Acute cellular rejection is histologically characterized by lymphocyte infiltration leading to myocardial necrosis and local edema (4, 16). We have previously demonstrated that the rejection severity is reflected by the LVGLS magnitude (7). The mechanism and link between acute rejection and LVGLS are not fully clarified but may be explained by the longitudinal orientation of the sub-endomyocardial fibers that is believed to be particularly sensitive to ischemia, edema, and fibrosis. Nt-ProBNP is a well-known heart failure marker reflecting LV filling pressures and wall stress (17). During rejection, the Nt-ProBNP level may increase due to a direct myocyte damage and/or a secondary increase following the development of myocardial dysfunction and elevated filling pressures. Thus, Nt-ProBNP has been demonstrated a valuable tool for rejection evaluation (11, 12). It is well known that severe rejection episodes lead to myocyte necrosis. Therefore, it is reasonable to expect that high-sensitivity cardiac Troponins would be elevated as seen in other myocardial inflammatory diseases with myocyte necrosis such as myocarditis (18) and cardiac sarcoidosis (19). By combining LVGLS, Troponin T, and Nt-ProBNP, we obtain a non-invasive model that could potentially have a central role in graft surveillance in relation to acute rejection by reflecting the degree of myocardial edema, myocyte necrosis, and inflammation. Potentially, such a model could be used as gatekeeper to invasive biopsies by ruling in and out patients with respect to rejection surveillance. The results of the present study supported our hypothesis. Thus, LVGLS, Troponin T, and Nt-ProBNP were all significantly correlated with rejection degree. However, the sensitivity and specificity for each variable with respect to rejection were at most moderate using fixed cutoff points. This is expectable, as LVGLS, Troponin T, and Nt-ProBNP are all affected by other factors than rejection such as transport damage, the surgical trauma, renal dysfunction, elevated filling pressure, endomyocardial fibrosis, and development of cardiac allograft vasculopathy. These confounders are important and result in a wide range of individual values for LVGLS, Troponin T, and Nt-ProBNP, especially in the early phase after HTx. We previously demonstrated that LVGLS magnitude increases significantly during the first 3 months after HTx (20). Therefore, caution should be taken using specific cutoff points for rejection suspicion in this period.

In the serial interpretation of data, we noted a significant decline in LVGLS magnitude and increases in Troponin T and Nt-ProBNP during ≥2R rejection. However, the sensitivity was at most moderate using these parameters for rejection assessment. This may be explained by the natural changes in LVGLS, Troponin T, and Nt-ProBNP in the first months after HTx. Thus, in the early phase after HTx, the rejection-induced LVGLS, Troponin T, and Nt-ProBNP affection must be substantial in order for the model to raise suspicion for acute rejection. Therefore, the majority of cases with biopsy-proven rejection (≥2R) but no sudden drop in graft function were in the first months after HTx. Furthermore, 25% of cases with ≥2R rejection but no sudden drop in graft function were taken within 2 weeks from a treated rejection episode at which point the graft function was already affected by acute rejection. Importantly, not all asymptomatic 2R rejection episodes need intensive intravenous methylprednisolone treatment (21). We speculate that cases with biopsy-proven 2R rejection but without affected LVGLS, Troponin T, and Nt-ProBNP do not need intravenous methylprednisolone treatment. In our study, we only omitted intravenous methylprednisolone treatment in three cases with biopsy-proven rejection but no drop in graft function. All had a favorable prognosis during follow-up, but larger studies are needed to evaluate the safety of omitted intravenous methylprednisolone treatment in cases without sudden drop in graft function.

In our study, we noted a strong negative predictive value of the non-invasive model combining LVGLS, Troponin T, and Nt-ProBNP assessment. Furthermore, the model identified other important hazards such as cardiac allograft vasculopathy and potential false negative biopsies. However, the proposed non-invasive model missed several ≥2R rejections, which could be reduced by use of the model only after the first 3 months and patients with a recent treatment demanding rejection. Importantly, our non-invasive rejection model was based on predefined cutoff values. In clinical practice, evaluation of trends may be useful as a gradual reduction in LVGLS magnitude or increase in Troponin T and Nt-ProBNP over time should raise suspicion of rejection. Thus, we noted a significant relation between the magnitude of LVGLS, Troponin T, and Nt-ProBNP change and the risk of treatment demanding rejection. Potentially, the non-invasive model evaluating a sudden drop in graft function may significantly reduce the number of biopsies and the associated risk of pneumothorax, cardiac tamponade, lung embolism, tricuspid valve damage, and procedure-related death. Furthermore, the model could be cost effective, but there is a need of a randomized trial evaluating the outcome and safety of non-invasive rejection monitoring LVGLS, Troponin T, and Nt-ProBNP versus traditional biopsy surveillance.

### Limitations

This study is a single-center experience in a small cohort of patients. However, we used repeated measurements with a predefined standard protocol for rejection monitoring by speckle tracking imaging and serological cardiac markers. We have previously reported low interobserver variation for LVGLS (7). However, the interobserver variation for the histological rejection graduation is unknown. Thus, it is possible that some episodes with a sudden drop in graft function but no 2R rejection were false negative and that some episodes with 2R rejection but no sudden drop in graft function were false positive.

## Conclusions

Treatment demanding rejection was observed in only 3% of biopsies. Cases with ≥2R rejection had a lower LVGLS magnitude and higher Troponin T and Nt-ProBNP levels than cases without 2R rejection. In addition, significant changes in these parameters were observed during the course of grade 2R rejection compared to pre-rejection levels. A non-invasive model combining changes in LVGLS, Troponin T, or Nt-ProBNP showed excellent negative predictive value and may be used as a cost-effective non-invasive gatekeeper to the invasive biopsies after HTx.

## Data Availability Statement

The raw data supporting the conclusions of this article will be made available by the authors, without undue reservation.

## Ethics Statement

Ethical review and approval was not required for the study in accordance with the local legislation and institutional requirements. The regional data protection agency approved the study. Written informed consent for participation was not required for this study in accordance with the national legislation and the institutional requirements.

## Author Contributions

Designed the study: all authors. Data collection: TC, NF, FO, HE. Data management: TC. Writing of the paper: all authors. All authors contributed to the article and approved the submitted version.

## Conflict of Interest

The authors declare that the research was conducted in the absence of any commercial or financial relationships that could be construed as a potential conflict of interest.

## Publisher’s Note

All claims expressed in this article are solely those of the authors and do not necessarily represent those of their affiliated organizations, or those of the publisher, the editors and the reviewers. Any product that may be evaluated in this article, or claim that may be made by its manufacturer, is not guaranteed or endorsed by the publisher.

## References

[1] Lund LH Edwards LB Kucheryavaya AY Benden C Dipchand AI Goldfarb S. The Registry of the International Society for Heart and Lung Transplantation: Thirty-Second Official Adult Heart Transplantation Report–2015; Focus Theme: Early Graft Failure. J Heart Lung Transpl (2015) 34:1244–54. doi: 10.1016/j.healun.2015.08.003 26454738

[2] Wu GW Kobashigawa JA Fishbein MC Patel JK Kittleson MM Reed EF. Asymptomatic Antibody-Mediated Rejection After Heart Transplantation Predicts Poor Outcomes. J Heart Lung Transpl (2009) 28:417–22. doi: 10.1016/j.healun.2009.01.015 PMC3829690 19416767

[3] Raichlin E Edwards BS Kremers WK Clavell AL Rodeheffer RJ Frantz RP. Acute Cellular Rejection and the Subsequent Development of Allograft Vasculopathy After Cardiac Transplantation. J Heart Lung Transpl (2009) 28:320–7. doi: 10.1016/j.healun.2009.01.006 19332257

[4] Patel JK Kittleson M Kobashigawa JA. Cardiac Allograft Rejection. Surgeon (2011) 9:160–7. doi: 10.1016/j.surge.2010.11.023 21550522

[5] Bader FM Islam N Mehta NA Worthen N Ishihara S Stehlik J. Noninvasive Diagnosis of Cardiac Allograft Rejection Using Echocardiography Indices of Systolic and Diastolic Function. Transplant Proc (2011) 43:3877–81. doi: 10.1016/j.transproceed.2011.09.039 22172863

[6] From AM Maleszewski JJ Rihal CS. Current Status of Endomyocardial Biopsy. Mayo Clin Proc (2011) 86:1095–102. doi: 10.4065/mcp.2011.0296 PMC3203000 22033254

[7] Clemmensen TS Logstrup BB Eiskjaer H Poulsen SH. Changes in Longitudinal Myocardial Deformation During Acute Cardiac Rejection: The Clinical Role of Two-Dimensional Speckle-Tracking Echocardiography. J Am Soc Echocardiogr (2015) 28:330–9. doi: 10.1016/j.echo.2014.10.015 25499656

[8] Kato TS Oda N Hashimura K Hashimoto S Nakatani T Ueda HI. Strain Rate Imaging Would Predict Sub-Clinical Acute Rejection in Heart Transplant Recipients. Eur J Cardiothorac Surg (2010) 37:1104–10. doi: 10.1016/j.ejcts.2009.11.037 20031437

[9] Sehgal S Blake JM Sommerfield J Aggarwal S. Strain and Strain Rate Imaging Using Speckle Tracking in Acute Allograft Rejection in Children With Heart Transplantation. Pediatr Transpl (2015) 19:188–95. doi: 10.1111/petr.12415 25532819

[10] Mingo-Santos S Monivas-Palomero V Garcia-Lunar I Mitroi CD Goirigolzarri-Artaza J Rivero B. Usefulness of Two-Dimensional Strain Parameters to Diagnose Acute Rejection After Heart Transplantation. J Am Soc Echocardiogr (2015) 28:1149–56. doi: 10.1016/j.echo.2015.06.005 26165446

[11] Arnau-Vives MA Almenar L Hervas I Osa A Martinez-Dolz L Rueda J. Predictive Value of Brain Natriuretic Peptide in the Diagnosis of Heart Transplant Rejection. J Heart Lung Transpl (2004) 23:850–6. doi: 10.1016/j.healun.2003.08.005 15261180

[12] Damodaran A Dardas T Wu AH Dyke DB Hummel SL Cowger JA. Changes in Serial B-Type Natriuretic Peptide Level Independently Predict Cardiac Allograft Rejection. J Heart Lung Transpl (2012) 31:708–14. doi: 10.1016/j.healun.2012.02.014 22502810

[13] Fitzsimons S Evans J Parameshwar J Pettit SJ. Utility of Troponin Assays for Exclusion of Acute Cellular Rejection After Heart Transplantation: A Systematic Review. J Heart Lung Transpl (2018) 37:631–8. doi: 10.1016/j.healun.2017.12.008 29426716

[14] Stewart S Winters GL Fishbein MC Tazelaar HD Kobashigawa J Abrams J. Revision of the 1990 Working Formulation for the Standardization of Nomenclature in the Diagnosis of Heart Rejection. J Heart Lung Transpl (2005) 24:1710–20. doi: 10.1016/j.healun.2005.03.019 16297770

[15] Cerqueira MD Weissman NJ Dilsizian V Jacobs AK Kaul S Laskey WK. American Heart Association Writing Group on Myocardial Segmentation and Registration for Cardiac Imaging. Standardized Myocardial Segmentation and Nomenclature for Tomographic Imaging of the Heart. A Statement for Healthcare Professionals From the Cardiac Imaging Committee of the Council on Clinical Cardiology of the American Heart Association. Circulation (2002) 105:539–42. doi: 10.1161/hc0402.102975 11815441

[16] Puleo JA Aranda JM Weston MW Cintron G French M Clark L. Noninvasive Detection of Allograft Rejection in Heart Transplant Recipients by Use of Doppler Tissue Imaging. J Heart Lung Transpl (1998) 17:176–84. 9513856

[17] Ponikowski P Voors AA Anker SD Bueno H Cleland JG Coats AJ. Authors/Task Force Members, Document Reviewers. 2016 ESC Guidelines for the Diagnosis and Treatment of Acute and Chronic Heart Failure: The Task Force for the Diagnosis and Treatment of Acute and Chronic Heart Failure of the European Society of Cardiology (ESC). Developed With the Special Contribution of the Heart Failure Association (HFA) of the ESC. Eur J Heart Fail (2016) 18:891–975. doi: 10.1002/ejhf.592 27207191

[18] Kindermann I Barth C Mahfoud F Ukena C Lenski M Yilmaz A. Update on Myocarditis. J Am Coll Cardiol (2012) 59:779–92. doi: 10.1016/j.jacc.2011.09.074 22361396

[19] Kandolin R Lehtonen J Airaksinen J Vihinen T Miettinen H Kaikkonen K. Usefulness of Cardiac Troponins as Markers of Early Treatment Response in Cardiac Sarcoidosis. Am J Cardiol (2015) 116:960–4. doi: 10.1016/j.amjcard.2015.06.021 26209113

[20] Clemmensen TS Logstrup BB Eiskjaer H Poulsen SH. Serial Changes in Longitudinal Graft Function and Implications of Acute Cellular Graft Rejections During the First Year After Heart Transplantation. Eur Heart J Cardiovasc Imaging (2016) 17:184–93. doi: 10.1093/ehjci/jev133 PMC4882878 26034093

[21] Costanzo MR Dipchand A Starling R Anderson A Chan M Desai S. International Society of Heart and Lung Transplantation Guidelines. The International Society of Heart and Lung Transplantation Guidelines for the Care of Heart Transplant Recipients. J Heart Lung Transpl (2010) 29:914–56. doi: 10.1016/j.healun.2010.05.034 20643330

