# Supplementary material for: Assessment of Acute Rejection by Global Longitudinal Strain and Cardiac Biomarkers in Heart-Transplanted Patients
Source: Front Immunol. 2022 Mar 25;13:841849. doi: 10.3389/fimmu.2022.841849 (PMC8990963; doi:10.3389/fimmu.2022.841849)
Supplement: Supplementary file 1 [file Table_1.docx]

| Patient | ACR grade | Time since HTx (months) | Treated with MP | CAV (ISHLT class 0-3) | Arrhythmia induced graft failure | Previous 2R ACR (number) | Later 2R ACR (number) | Increase in creatinine >30% | Sudden CVD  During follow-up |
| --- | --- | --- | --- | --- | --- | --- | --- | --- | --- |
| 1 | 1 | 2 | 0 | 0 | 0 | 0 | 1 | 0 | 0 |
| 2 | 0 | 18 | 0 | 1 | 0 | 1 | 0 | 0 | 0 |
| 3 | 1 | 6 | 0 | 1 | 0 | 0 | 0 | 0 | 0 |
| 4 | 1 | 6 | 1 | 0 | 0 | 0 | 1 | 0 | 1 |
| 4 | 0 | 8 | 0 | 0 | 0 | 1 | 1 | 0 | 1 |
| 5 | 1 | 29 | 0 | 0 | 1 | 1 | 0 | 0 | 0 |
| 6 | 0 | 2 | 0 | 0 | 0 | 0 | 1 | 0 | 0 |
| 7 | 0 | 6 | 0 | 0 | 0 | 0 | 0 | 0 | 0 |
| 7 | 0 | 24 | 0 | 0 | 0 | 0 | 0 | 0 | 0 |
| 8 | 1 | 1 | 0 | 0 | 0 | 1 | 0 | 0 | 0 |
| 9 | 0 | 1 | 0 | 0 | 0 | 0 | 0 | 1 | 0 |
| 10 | 0 | 6.8 | 0 | 1 | 0 | 1 | 0 | 0 | 0 |
| 11 | 1 | 24 | 0 | 2 | 0 | 0 | 0 | 0 | 1 |
| 12 | 0 | 21 | 0 | 0 | 0 | 0 | 0 | 0 | 0 |
| 13 | 1 | 8 | 0 | 0 | 0 | 0 | 0 | 0 | 0 |
| 14 | 1 | 6 | 0 | 0 | 0 | 0 | 0 | 0 | 0 |
| 15 | 1 | 19 | 0 | 1 | 0 | 1 | 0 | 0 | 0 |
| 16 | 1 | 6 | 0 | 0 | 0 | 0 | 0 | 0 | 0 |
| 17 | 1 | 1 | 0 | 0 | 0 | 1 | 1 | 1 | 0 |
| 18 | 1 | 2 | 0 | 0 | 0 | 0 | 0 | 0 | 0 |
| 19 | 1 | 12 | 0 | 1 | 0 | 1 | 0 | 0 | 1 |
| 20 | 1 | 9 | 1 | 1 | 0 | 2 | 0 | 0 | 1 |
| 20 | 1 | 22 | 0 | 2 | 0 | 2 | 0 | 0 | 1 |
| 21 | 1 | 0.5 | 0 | 0 | 0 | 0 | 3 | 1 | 0 |

Supplementary table 1: Sudden drop in graft function but no biopsy detected acute rejection (≥2R)

ACR = acute cellular rejection, HTx = heart transplantation, MP = methyl prednisolone, CAV = cardiac allograft vasculopathy, ISHLT = International Society of Heart and Lung Transplantation, CVD = cardio-vascular death.
