# Supplementary material for: Assessment of Acute Rejection by Global Longitudinal Strain and Cardiac Biomarkers in Heart-Transplanted Patients
Source: Front Immunol. 2022 Mar 25;13:841849. doi: 10.3389/fimmu.2022.841849 (PMC8990963; doi:10.3389/fimmu.2022.841849)
Supplement: Supplementary file 2 [file Table_2.docx]

Supplementary table 2: No sudden drop in graft function but biopsy detected acute rejection (≥2R)

| Patient | ACR grade | Treated rejection < 2 weeks prior biopsy | Time since HTx (months) | Treated with MP | CAV (ISHLT class 0-3) | Arrhythmia induced graft failure | Previous 2R ACR (number) | Later 2R ACR (number) | Increase in creatinine >30% | Sudden CVD during follow-up |
| --- | --- | --- | --- | --- | --- | --- | --- | --- | --- | --- |
| 1 | 2 | 0 | 1 | 1 | 0 | 0 | 1 | 0 | 0 | 0 |
| 2 | 2 | 0 | 1 | 1 | 0 | 0 | 0 | 1 | 0 | 0 |
| 2 | 2 | 1 | 1.5 | 1 | 0 | 0 | 1 | 0 | 0 | 0 |
| 3 | 2 | 0 | 12 | 0 | 0 | 0 | 0 | 0 | 0 | 0 |
| 4 | 2 | 0 | 3 | 0 | 0 | 0 | 0 | 0 | 0 | 0 |
| 5 | 2 | 0 | 1 | 1 | 0 | 0 | 0 | 0 | 0 | 0 |
| 6 | 2 | 0 | 0.5 | 1 | 0 | 0 | 0 | 1 | 0 | 0 |
| 6 | 2 | 1 | 0.7 | 1 | 0 | 0 | 1 | 0 | 0 | 0 |
| 7 | 2 | 0 | 5 | 1 | 0 | 0 | 0 | 0 | 0 | 0 |
| 8 | 2 | 0 | 3 | 1 | 0 | 0 | 0 | 0 | 0 | 0 |
| 9 | 2 | 1 | 1 | 1 | 0 | 0 | 1 | 0 | 0 | 0 |
| 10 | 2 | 0 | 0.7 | 1 | 0 | 0 | 0 | 1 | 0 | 0 |
| 11 | 2 | 0 | 2 | 1 | 0 | 0 | 0 | 0 | 0 | 1 |
| 12 | 2 | 0 | 2 | 1 | 0 | 0 | 0 | 1 | 0 | 1 |
| 12 | 2 | 1 | 4 | 1 | 0 | 0 | 1 | 0 | 0 | 1 |
| 13 | 2 | 0 | 1 | 1 | 0 | 0 | 0 | 1 | 0 | 0 |
| 13 | 2 | 0 | 15 | 1 | 0 | 0 | 1 | 0 | 0 | 0 |
| 14 | 2 | 0 | 5 | 1 | 0 | 0 | 0 | 0 | 0 | 0 |
| 15 | 2 | 0 | 17 | 1 | 0 | 0 | 0 | 1 | 0 | 0 |
| 15 | 2 | 1 | 17.5 | 0 | 0 | 0 | 1 | 0 | 0 | 0 |

ACR = acute cellular rejection, AMR = antibody mediated rejection, HTx = heart transplantation, MP = methyl prednisolone, CAV = cardiac allograft vasculopathy, ISHLT = International Society of Heart and Lung Transplantation, CVD = cardio-vascular death.
